# Antimicrobial Activity of Soil Clostridium Enriched Conditioned Media Against *Bacillus mycoides*, *Bacillus cereus*, and *Pseudomonas aeruginosa*

**DOI:** 10.3389/fmicb.2020.608998

**Published:** 2020-12-04

**Authors:** Amila Srilal Nawarathna Weligala Pahalagedara, Steve Flint, Jon Palmer, Arvind Subbaraj, Gale Brightwell, Tanushree Barua Gupta

**Affiliations:** ^1^Food Assurance team, AgResearch Ltd., Hopkirk Research Institute, Massey University, Palmerston North, New Zealand; ^2^School of Food and Advanced Technology, Massey University, Palmerston North, New Zealand; ^3^Proteins and Metabolites team, AgResearch Ltd., Lincoln Research Centre, Lincoln, New Zealand

**Keywords:** soil, non-targeted metabolomics, antimicrobial compounds, strict anaerobe, *Clostridium* spp.

## Abstract

The rise of antimicrobial resistant bacteria has fast-tracked the exploration for novel antimicrobial compounds. Reports on antimicrobial producing soil anaerobes such as *Clostridium* spp. are very limited. In the present study, the antimicrobial activity of soil *Clostridium* enriched conditioned/spent media (CMs) against *Bacillus mycoides*, *Bacillus cereus* and *Pseudomonas aeruginosa* was assessed by turbidimetric growth inhibition assay. Our results highlighted the antimicrobial potential of soil *Clostridium* enriched conditioned media against pathogenic and spoilage bacteria. Farm 4 soil conditioned medium (F4SCM) demonstrated a greater growth inhibition activity against all three tested microorganisms in comparison to other soil conditioned media. Non-targeted metabolite profiling of all soil conditioned media revealed distinctive polar and intermediate-polar metabolites in F4SCM, consistent with its strong antimicrobial property. Moreover, 539 significantly abundant metabolites including some unique features were detected in F4SCM suggesting its substantial and specialized chemical diversity. This study putatively identified seven significantly high metabolites in F4SCM; 3-hydroxyphenylacetic acid, γ-aminobutyric acid, creatine, tryptamine, and 2-hydroxyisocaproic acid. Tryptamine and 2-hydroxyisocaproic acid were previously reported to have antimicrobial properties. The present study shows that soil *Clostridium* spp. are a promising group of bacteria producing metabolites with antimicrobial activity and provides future prospects for clostridial antimicrobial discovery within their metabolic diversity.

## Introduction

The global spread of antimicrobial resistant bacteria and the emerging consumer trend for natural food preservatives, emphasize the need of exploring novel and natural antimicrobial compounds (Santos-Sanchez et al., 2017; D’Andrea et al., 2019). In this context, microbial diversity in many environments has been a great interest to explore new antimicrobials (Mullis et al., 2019). *Clostridium* spp., ubiquitously present in extreme, complex and dynamic environments such as soil and human and animal gut have received limited attention in bioactive compound discovery. Conceivably, their secondary metabolism has been evolved giving rise to various adaptative mechanisms to survive and proliferate in challenging environments (Morris, 1994). Therefore, the synthesis of bioactive secondary metabolites, including antimicrobials, may help the survival of *Clostridium* spp. in challenging and stressed environments. Pathogenicity of this genus has received more interest, even though most of *Clostridium* spp. are saprophytes and not involved in a disease process (Liu, 2011). Recent genome mining studies have identified the genetic potential of *Clostridium* spp. for antimicrobial biosynthesis (Tracanna et al., 2017). Analysis of publicly available genomes revealed the presence of biosynthesis gene clusters (BGCs) tentatively encoding for non-ribosomal peptide synthetases (NRPSs) and polyketide synthases (PKSs) in the members of the genus *Clostridium* (Letzel et al., 2013). It was also found that environmental *Clostridium* isolates from sources such as soil possess a higher number of biosynthetic gene clusters (BGCs) and higher variability in cluster composition than human associated isolates (Letzel et al., 2013). This emphasizes the prospect of novel antimicrobial discovery from soil *Clostridium* species. Limited studies conducted thus far with *Clostridium* spp. have identified several novel clostridial antimicrobials such as closthioamide, clostrubin, and clostrindolin and some of these compounds are known to have novel structures and/or mechanisms of action effective against multi-drug resistant bacteria (Lincke et al., 2010; Pidot et al., 2014; Schieferdecker et al., 2019). In this scenario, investigating the antimicrobial potential of soil *Clostridium* spp. against significant bacteria of human health and food quality is of great interest.

*Pseudomonas aeruginosa* is a well-known opportunistic pathogen that can be multi-drug-resistant and may restrict the available treatment options for its infections, making it a critical public healthcare concern (Bassetti et al., 2018). *Bacillus cereus* and *Bacillus mycoides* are ubiquitous and commonly associated with compromising food quality and human pathogenicity. *B. cereus* is recognized as a significant cause of food poisoning and commonly associated with contaminated milk, cereals and various other foods, whereas, *B. mycoides* are mainly associated with food spoilage and their food poisoning potential appears to be low (Prüß et al., 1999). These are a few examples of bacteria that could be targeted by novel control measures due to their adverse impact on human health and food systems.

Our previous studies demonstrated that when farm environment samples were added and enriched in cooked meat broth under anaerobic growth conditions, only *Clostridium* spp. could thrive, inhibiting the growth of other facultative anaerobic microorganisms present in samples. Preliminary studies showed that these *Clostridium* enriched media/conditioned media (CMs) possessed antimicrobial activity against certain bacteria (internal communication). In the present study, antimicrobial potential of soil *Clostridium* enriched conditioned media against three bacterial species of significant health and food quality concern was investigated. The soil CM with the highest antimicrobial potential was further characterized and *Clostridium* spp. from soil sample were isolated and identified. Non-targeted metabolomics analysis was carried out to provide further insight into metabolite profiles after the enrichment and their prospective antimicrobial properties.

## Materials and Methods

### Bacterial Strains and Growth Conditions

*Bacillus mycoides* ATCC 6462, *Bacillus cereus* NZRM 5, and *Pseudomonas aeruginosa* ATCC 25668 were obtained from environmental science and research (ESR), New Zealand. All frozen glycerol stocks were revived on sheep blood agar (SBA) plates (Fort Richard Laboratories, New Zealand) and incubated at 35°C overnight. All microorganisms were further grown in tryptic soy broth (TSB) and incubated at 35°C overnight as required for experiments.

### Preparation of Conditioned Media (CMs)

Farm soil samples were collected from paddocks of five bovine dairy farms in the Manawatu region, New Zealand during winter and stored at −20°C until use. Each of the soil samples (15 g) were suspended in sterile 50 mL butterfield’s diluent (0.31 mM monobasic potassium phosphate, pH adjusted to 7.2 ± 0.1) and blended using a laboratory stomacher (Seward Stomacher 400, United Kingdom) at high speed for 2 min. Suspensions were centrifuged at 10,000 × g for 1 h at 4°C. The pellets were re-suspended in 5 mL butterfield’s diluent and heated at 80°C for 10 min in a water bath (Grant, United Kingdom) to kill vegetative cells (Gupta and Brightwell, 2017).

Cooked meat glucose starch medium (CMGS; 27 mL) (Fort Richard Laboratories, New Zealand) supplemented with yeast extract (0.0005%), hemin (0.1%), and vitamin K (1%) was inoculated with a 3 mL aliquot of the heated sample suspension and incubated in an anaerobic chamber (Don Whitley Scientific, United Kingdom) at 35°C for 48 h. After incubation, the broth suspensions were centrifuged at 10,000 × g for 40 min at 4°C and the supernatants (conditioned media) were filter sterilized using 0.22 μm polyvinylidene fluoride (PVDF) filter membrane (Millipore, Ireland). Sterile conditioned medium from each sample was aliquoted and stored frozen at −20°C until use. The sterility of conditioned medium (CM) was verified by plating 100 μL of filter sterilized sample on SBA plates. Farm 1 soil conditioned medium was denoted as F1SCM and the rest as F2SCM, F3SCM, F4SCM, and F5SCM.

### Microplate Turbidimetric Growth Inhibition Assay

The bacterial growth inhibition was measured by determining the optical density (OD) of each indicator bacterial culture at 595 nm periodically using Multiskan GO microplate spectrophotometer with Skanlt software version 3.2 (Thermo Fisher Scientific, United States) as described by Vijayakumar and Muriana (2015) with some modifications. Briefly, overnight bacterial culture was diluted to achieve ∼ 1 × 10^7^ CFU/mL cell density and the adjusted bacterial culture (50 μL) was added to CMGS broth (50 μL) and conditioned medium (100 μL) in a 96-well flat bottom microtiter plate (Thermo Fisher Scientific, Denmark) and covered with a Breathe-Easy^®^ sealing membrane (Diversified Biotech, United States). The plate was incubated in a microplate spectrophotometer at 35°C and bacterial growth in each well was assessed by measuring the OD at 595 nm for 24 h. The optical density values were plotted against time to obtain bacterial growth curves. Nisin (45 μM) and Butterfield’s diluent were used as the positive control and untreated control, respectively. Corresponding blanks (growth medium, conditioned media, and nisin) were used for background correction.

### Effect of Heat, pH and Protease Enzyme on the Antimicrobial Activity of Farm 4 Soil Conditioned Medium

F4SCM was subjected to various heat treatments; 50, 70, 80, 90°C for 10 min and 90°C for 20 min and the antimicrobial activity following these treatments was assessed by the microplate turbidimetric growth inhibition assay using all three indicator micro-organisms. The influence of pH and protease enzyme on the F4SCM’s antimicrobial activity was determined as described by Lasik-Kurdyś and Sip (2019) with minor modifications. Briefly, the pH of F4SCM was adjusted to 2, 4, 6, 8, 10, and 12 using 5M hydrochloric acid (HCl) and 5M sodium hydroxide (NaOH) and incubated at room temperature (25°C) for 60 min. Samples were re-adjusted to pH 7.2 ± 0.2 and the residual antimicrobial activity was determined by the turbidimetric growth inhibition assay. To investigate the susceptibility of F4SCM to protease enzyme, F4SCM was treated with protease (Sigma-Aldrich Co., United States) at a final concentration of 1 mg/mL and incubated at 37°C for 60 min. The residual antimicrobial activity was measured against all three indicator microorganisms using the microplate turbidimetric growth inhibition assay. To ensure that 1 mg/mL protease enzyme has no effect on the growth of bacteria, it was used alone as a control.

### Isolation of *Clostridium* spp. From Farm 4 Soil

*Clostridium* spp. were isolated from F4SCM using the method described by Gupta and Brightwell (2017) with minor modifications. Briefly, enriched culture was centrifuged at 10,000 × g for 20 min and the cell pellet was re-suspended in 5 mL of Butterfield’s diluent. Ten-fold serial dilutions of the enriched cell suspension was plated on Shahidi Ferguson Perfringens (SFP) base agar containing 50% egg yolk emulsion, kanamycin (12 μg/mL) and polymyxin B (Becton Dickinson, France) and overlaid with SFP agar with no supplements followed by incubation at 35°C for 24 h under anaerobic conditions. Individual colonies were picked and sub-cultured on fresh SBA plates and incubated anaerobically at 35°C for 24 h. Each isolate was inoculated into thioglycolate broth (Fort Richard Laboratories, New Zealand) for DNA isolation and further genomic analysis.

### Identification of Farm 4 Soil Isolates by 16S rRNA Gene Sequence Analysis

Genomic DNA from each isolate was extracted using a genomic DNA extraction kit (Roche diagnostics, Germany) according to the manufacturer’s instructions. The 16S rRNA gene sequences were amplified from extracted DNA using primers, forward: PA 5′ –AGA GTT TGA TCC TGG CTC AG-3′ (Invitrogen) and reverse: PH^∗^ 5′- AAG GAG GTG ATC CAG CCG CA-3′ (Invitrogen) as described by Böddinghaus et al. (1990). Amplification was carried out in a thermal cycler (Eppendorf Mastercycler pro, Germany) using the following conditions: initial denaturation at 93°C for 3 min, followed by 30 individual cycles consisting of denaturation at 92°C for 1 min, annealing at 55°C for 1 min and extension at 72°C for 1 min. The final products were extended at 72°C for 2 min and stored at 4°C. Amplified products were separated by gel electrophoresis using 0.8 agarose gel, stained with ethidium bromide and visualized by an UV transilluminator. The PCR products were purified by QIAquick PCR purification kit (Qiagen^®^, Germany) and sequenced at Massey University genome service, New Zealand. All the 16S rRNA gene sequences were processed using Geneious software version 10 (Kearse et al., 2012). The consensus sequences obtained were compared with the RDP 16S rRNA sequences database^1^ to identify closest taxonomically described species.

### Spiking of *Clostridium* Isolates in Sterile Soil

Farm 4 soil was autoclaved at 123°C for 30 min and cooled down to room temperature (25°C). Sterility of soil was confirmed by plating soil on SBA plates. All four *Clostridium* spp. identified from Farm 4 soil, were grown in TSB under anaerobic conditions at 35°C. Ten grams of sterile soil were spiked with the cocktail of all four isolates (200 μL of each) and mixed well. Spiked soil was then incubated anaerobically at 35°C for 48 h. Sterile soil alone was employed as negative control. CMs were prepared from spiked and sterile soil samples and their antimicrobial activities were measured using growth inhibition assay.

### Sample Preparation for Non-targeted Metabolomics

Five biological replicates of CMs were prepared from each soil sample collected from five farms. Extra-cellular polar and non-polar metabolites present in soil CMs were separated using liquid-liquid extraction. Briefly, conditioned medium (1 mL) was centrifuged at 11,900 x g for 10 min and the supernatant (200 μL) was mixed with 800 μL of pre-chilled chloroform:methanol (1:1, v/v) containing 1.6 mg/L of internal standards; d_5_-L-tryptophan, d_4_-citric acid, d_10_-leucine, d_2_-tyrosine, d_35_-stearic acid, d_5_-benzoic acid, ^13^C_2_-glucose, and d_7_-alanine. After mixing, 400 μL of Milli Q water (Milli-Q^®^, Germany) was also added. After 15 min of centrifugation at 11,900 x g, 200 μL of upper aqueous phase was taken and evaporated under a N_2_ stream at 30°C. Dried samples were reconstituted in 200 μL of acetonitrile:water (1:9, v/v) for C18 and acetonitrile:water (1:1, v/v) for hydrophilic interaction liquid chromatography (HILIC)–mass spectrometry analyses. Same procedure was followed for all CMs and CMGS, which was used as the negative control for comparing metabolite profiles.

### C18 and HILIC-MS Conditions, Analytical Procedure and Metabolomics Data Processing

For semi-polar compounds, 2 μL of metabolite extract was injected into a 100 mm x 2.1 mm Hypersil Gold C18 column with 1.9 μm particle size (Thermo Fisher Scientific, United States) and eluted over a 16 min gradient with a flow rate of 400 μL/min. The mobile phase was a mixture of Milli Q water with 0.1% formic acid (solvent A), and acetonitrile with 0.1% formic acid (solvent B). Gradient and other LC-MS conditions were set as described by Fraser et al. (2013). For polar compounds, HILIC conditions were set as described by Subbaraj et al. (2016). Two microliters of metabolite extract was injected into a 100 mm × 2.1 mm ZIC-pHILIC column with 5 μm particle size (Merk, Germany) and eluted over a 24 min gradient with a flow rate of 250 μL/min. Mobile phase solvent A was a mixture of acetonitrile with 0.1% formic acid and solvent B consisted of Milli Q water with 16 mM ammonium formate. C18 and HILIC column effluents were connected to the Exactive Orbitrap^TM^ (Thermo Fisher Scientific, United States) mass spectrometer with electrospray ionization. All samples were analyzed in negative and positive ionization modes. Pooled sample from all conditioned medium extracts and internal standards were used to maintain the data quality in both positive and negative mode analyzes. Samples were randomized prior to injection to avoid any systematic effects.

Non-targeted metabolomics data processing included eliminating background noise, discriminating signal from noise, correcting retention time shifts, and normalizing data for analytical drifts and sample variation to retain uniformity in the final data matrix. Raw data files were converted to mzML files using MSConvert function of ProteoWizard^TM^. Peak detection, retention time alignment, grouping and gap filling were performed using XCMS (Smith et al., 2006) and in-house scripts in R (Ihaka and Gentleman, 1996) with suitable parameters. The comparison between QC vs. blanks done using “diffreport” function of XCMS was used to identify non-significant *m/z* features (FDR *p*-value < 0.05) between the two groups. These features were removed from the QC vs. samples list of features as they correspond to background noise contributed by blanks. The QC vs. samples list was further cleaned by browsing all extracted ion chromatograms (EICs) generated by “diffreport” and removing *m/z* features representing background noise. The resulted data matrix was then normalized by a QC based robust LOESS signal correction (QC-RLSC) (Dunn et al., 2012) and run-order effects were evaluated before and after normalization. Relative standard deviation (RSD) of all *m/z* features in the QC was determined and features in the normalized data matrix with RSD > 0.3 were eliminated (Parsons et al., 2009).

### Metabolite Identification

Metabolomics Standard Initiative (MSI) has listed four metabolite identification levels in minimum reporting standards for metabolomics experiments (Sumner et al., 2007). According to these reporting standards, metabolite identification in the present study was performed at level two metabolite identification confidence, which was a comparison of parent mass (MS^1^) and diagnostic source-induced fragment/daughter ions (MS^2^) with corresponding matches in public spectral libraries. The online METLIN database and MassBank were used to putatively identify metabolite features by matching detected molecular mass data (m/z) with those from the database (10 ppm error tolerance).

### Statistical Analysis

All bacterial growth experiments were done in triplicate. The area under the experimental growth curve, which provides information on the overall bacterial growth under given growth conditions was computed using the R package “growthcurver” (Sprouffske and Wagner, 2016). Subsequent single factor Analysis of Variance (ANOVA) was performed to check the statistical significance of CM’s bacterial growth inhibition by comparing the growth in the presence and absence of CM. The values with *P* < 0.05 were considered statistically significant.

Metabolomics data were further subjected to multivariate and univariate analyses using MetaboAnalyst web tool^2^. Peak intensity data were checked for integrity and normalized using MetaboAnalyst’s normalization protocols (autoscaling) to improve the downstream statistical analysis. Principal component analysis (PCA) was performed using all detected metabolite features to find the directions of maximum variance in the dataset. F4SCM and CMGS data sets were used to calculate fold-change values (F4SCM/CMGS) and *p-*values using *t*-test, which were used to determine the statistical significance of each metabolite feature. Volcano plots were used to visualize both *p-*values and fold change values showing significantly discriminating metabolites in F4SCM and CMGS groups.

## Results

### Antimicrobial Activity of Soil Conditioned Media

**FIGURE 1:**
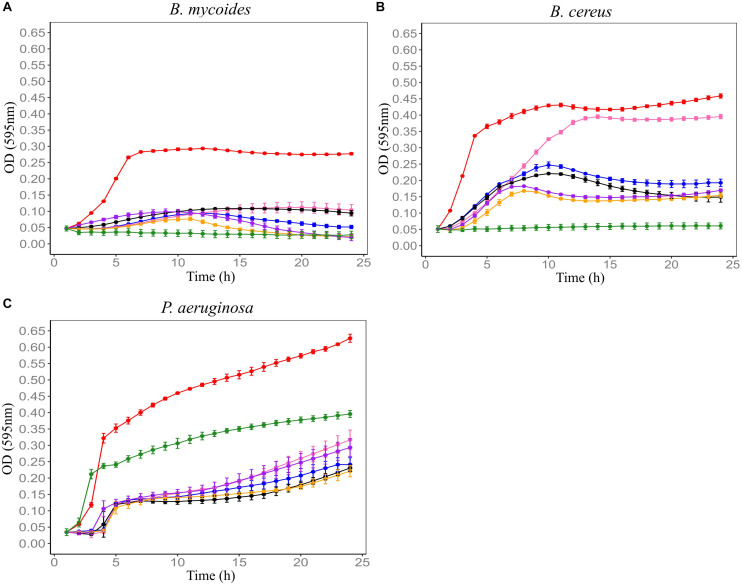
Effect of five soil conditioned media on the growth of *B. mycoides* ATCC 6462 **(A)**, *B. cereus* NZRM 5 **(B)**, and *P. aeruginosa* ATCC 25668 **(C)**. Bacteria were grown in the presence of butterfield’s diluent (red), F1SCM (Farm 1 soil conditioned media; blue), F2SCM (Farm 2 soil conditioned media; pink), F3SCM (Farm 3 soil conditioned media; black), F4SCM (Farm 4 soil conditioned media; orange), F5SCM (Farm 5 soil conditioned media; purple), and nisin (green) in the growth media (CMGS). Nisin (45 μM) and butterfield’s diluent served as positive and untreated control, respectively. Each curve represents the mean growth ± SD (*n* = 3). All treatments were significantly different from untreated control (*p* < 0.05).

In order to establish the CM with the best antimicrobial profiles, five soil CMs were tested for antimicrobial activity against *Bacillus mycoides* ATCC 6462, *Bacillus cereus* NZRM 5, and *Pseudomonas aeruginosa* ATCC 25668. Results showed that all five soil CMs significantly inhibited the growth of three tested microorganisms despite the differences in the level of activity. Out of all samples, F4SCM demonstrated the strongest antimicrobial activity while showing the best inhibition against *B. mycoides*. F1SCM, F3SCM, and F5SCM also significantly inhibited the growth of all three indicator microorganisms. Comparatively, F2SCM exhibited the least growth inhibition activity, particularly against *B. cereus* (Figure 1).

### Preliminary Characterization of Farm 4 Soil Conditioned Medium

**FIGURE 2:**
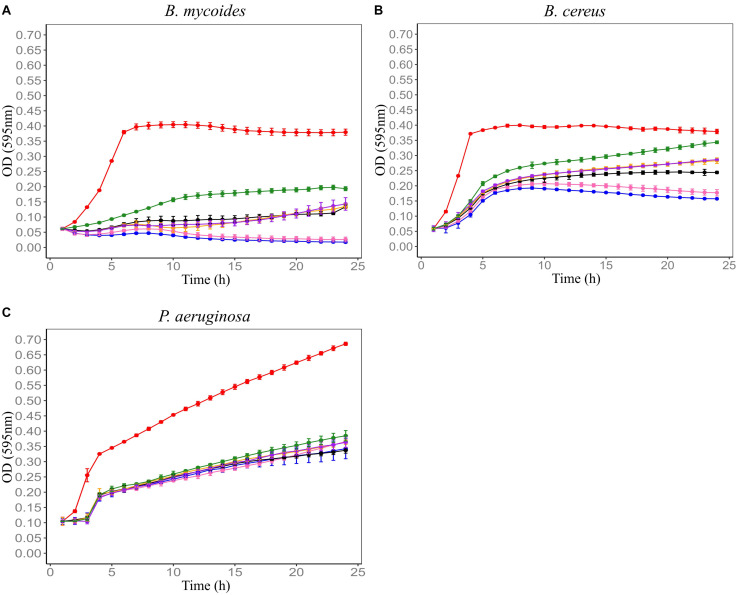
Influence of heat on the antimicrobial activity of Farm 4 soil conditioned medium (F4SCM) against *B. mycoides* ATCC 6462 **(A)**, *B. cereus* NZRM 5 **(B)**, and *P. aeruginosa* ATCC 25668 **(C)**. Bacteria were grown in the presence of butterfield’s diluent (red), F4SCM (blue), F4SCM treated at 50°C for 10 min (pink), F4SCM treated at 70°C for 10 min (black), F4SCM treated at 80°C for 10 min (orange), F4SCM treated at 90°C for 10 min (purple), and F4SCM treated at 90°C for 20 min (green) in the growth media (CMGS). Each curve represents the mean growth rate ± S.D (*n* = 3). Antimicrobial activity against *B. mycoides* and *B. cereus* was significantly decreased following heat treatment at 70°C or higher and there was no significant loss of activity against *P. aeruginosa* following various heat treatments (*p* < 0.05 vs. untreated control/F4SCM).

**FIGURE 3:**
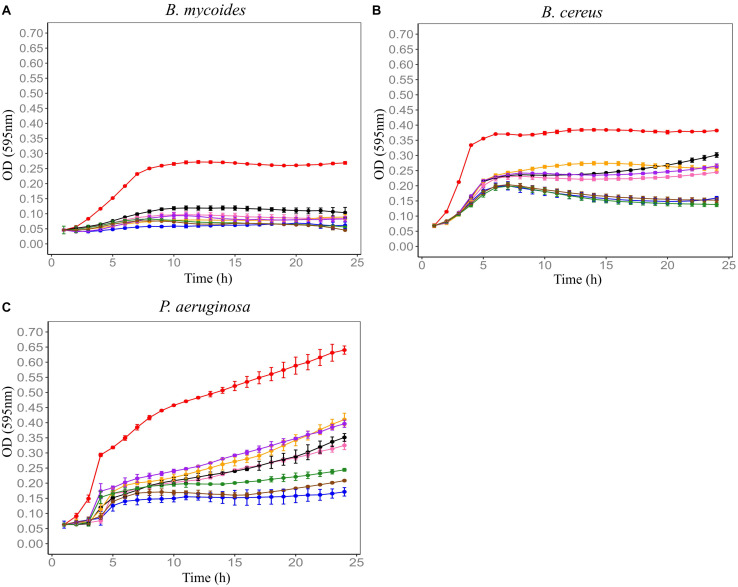
Influence of exposing Farm 4 soil conditioned medium (F4SCM) to different pH conditions on its antimicrobial activity against *B. mycoides* ATCC 6462 **(A)**, *B. cereus* NZRM 5 **(B)**, and *P. aeruginosa* ATCC 25668 **(C)**. Bacteria were grown in the presence of butterfield’s diluent (red), F4SCM (blue), F4SCM exposed to pH2 (orange), F4SCM exposed to pH4 (purple), F4SCM exposed to pH6 (green), F4SCM exposed to pH8 (brown), F4SCM exposed to pH10 (pink), F4SCM exposed to pH12 (black). Each curve represents the mean growth rate ± SD (*n* = 3). Significant loss of activity was observed at high acidic (pH 2 and 4) and basic pH values (pH 10 and 12) against all three indicator microorganisms (*p* < 0.05 vs. untreated control/F4SCM).

**FIGURE 4:**
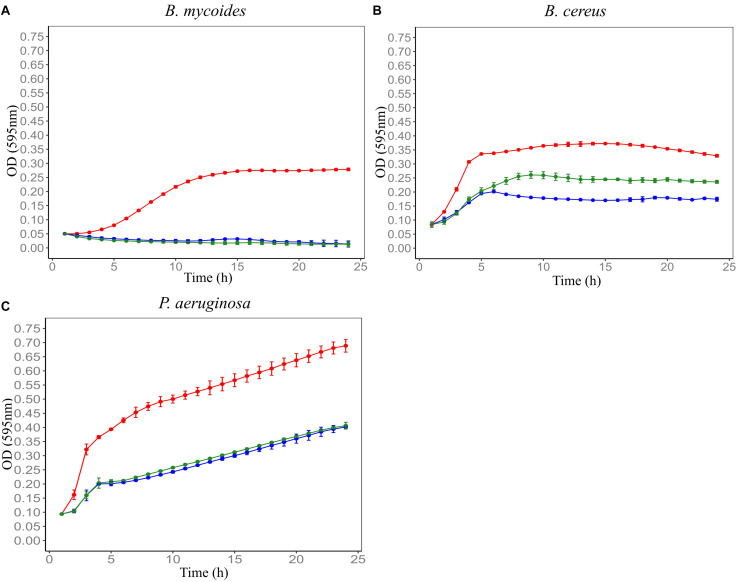
Effect of protease enzyme on the antimicrobial activity of Farm 4 soil conditioned medium (F4SCM) against *B. mycoides* ATCC 6462 **(A)**, *B. cereus* NZRM 5 **(B),** and *P. aeruginosa* ATCC 25668 **(C)**. Bacteria were grown in the presence of butterfield’s diluent (red), F4SCM (blue), and F4SCM treated with 1 mg/mL protease enzyme (green). Each curve represents the mean growth rate ± S.D (*n* = 3). Antimicrobial activity against *B. cereus* significantly changed after protease enzyme treatment (1 mg/mL) and there was no significant effect against *B. mycoides* and *P. aeruginosa* after protease enzyme treatment (*p* < 0.05 vs. untreated control/F4SCM).

F4SCM was found to be highly heat stable as it retained its antimicrobial activity against *P. aeruginosa* at 90°C up to 20 min. On the other hand, heat treatment impaired the antimicrobial activity against *B. cereus* in a temperature dependent manner. Its antimicrobial activity against *B. mycoides* was significantly decreased following heat treatment at 70°C or higher for 10 min (Figure 2). F4SCM treated with near neutral pH levels (pH 6 and 8) showed no significant change in the antimicrobial activity against *B. mycoides*. However, significant loss of activity was observed at highly acidic (pH 2 and 4) and basic pH values (pH 10 and 12). Following a similar pattern, the activity against *B. cereus* and *P. aeruginosa* was gradually decreased at lower and higher pH ranges (Figure 3). Antimicrobial activity of F4SCM against *B. mycoides* and *P. aeruginosa* remained stable after 1 mg/mL protease enzyme treatment. However, its activity against *B. cereus* significantly changed after protease enzyme treatment (Figure 4).

### *Clostridium* spp. Identified From Farm 4 Soil and Their Involvement in Antimicrobial Activity

In total, 18 bacterial isolates were recovered from Farm 4 soil spore enrichment and identified by 16S rRNA gene sequencing to the closest taxonomically described species. Results showed the sequences of all isolates to be similar to four different *Clostridium* species as shown in Table 1.

**TABLE 1:** Closely related *Clostridium* spp. isolated from Farm 4 soil.

**Isolate ID**	**Closest-related taxonomically described species**	**Maximum identity (%)**	**GenBank accession number**
FS01, FS2.1, FS05, FS06, FS07, FS08, FS09, FS16	*Paraclostridium bifermentans* (T); ATCC 638	98.1	AB075769
FS2.2, FS10, FS15, FS17	*Clostridium cadaveris* (T); JCM 1392	100	AB542932
FS03, FS14	*Clostridium glycolicum/Terrisporobacter glycolicus* (T); DSM 1288	95.6	X76750
FS4.1, FS4.2, FS12, FS13	*Clostridium senegalense* (T); JC122	98.2	JF824801

**FIGURE 5:**
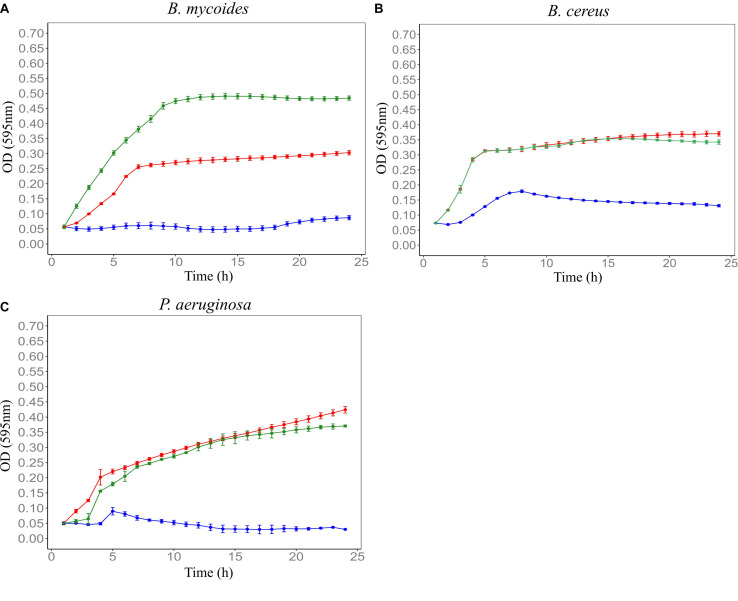
Involvement of *Clostridium* spp. isolated from Farm 4 soil in F4SCM’s antimicrobial activity. CMs were prepared from sterile Farm 4 soil (F4SCM_Sterile_) and Farm 4 soil spiked with *Clostridium* spp. (F4SCM_Spiked_) and evaluated their antimicrobial activities against *B. mycoides* ATCC 6462 **(A)**, *B. cereus* NZRM 5 **(B)**, and *P. aeruginosa* ATCC 25668 **(C)**. Bacteria were grown in the presence of butterfield’s diluent (red), F4SCM_Sterile_ (green), and F4SCM_Spiked_ (blue). Each curve represents the mean growth rate ± S.D (*n* = 3). F4SCM_Spiked_ significantly inhibited the growth of all three tested microorganisms while F4SCM_Sterile_ showed no antimicrobial activity (*p* < 0.05 vs. untreated control/butterfield’s diluent).

To determine the involvement of *Clostridium* spp. in antimicrobial activity of F4SCM, CMs prepared from sterile Farm 4 soil and sterile Farm 4 soil spiked with *Clostridium* isolates were investigated for their antimicrobial activity. CM prepared from sterile soil showed no growth inhibition against test microorganisms, while CM derived from soil spiked with *Clostridium* spp. significantly inhibited the growth of all test microorganisms (Figure 5).

### Non-targeted Metabolomic Profiling of Soil Conditioned Media

**FIGURE 6:**
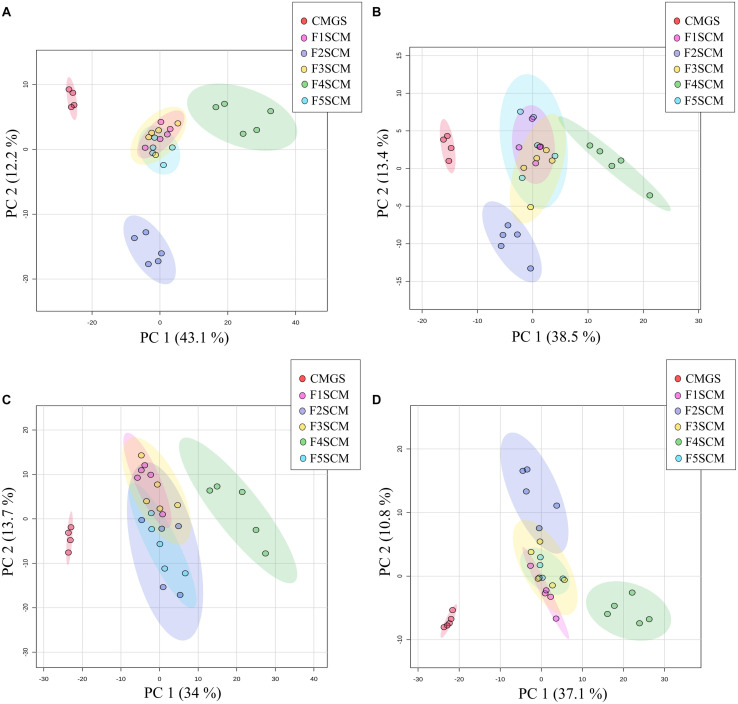
2D-PCA score plots of C18 negative **(A)**, C18 positive **(B)**, HILIC negative **(C)**, and HILIC positive **(D)** ionization mode data, showing differences in metabolite profiles of Farm 1–5 soil conditioned media. CMGS (Cooked meat glucose starch media supplemented with 0.0005% yeast extract, 0.1% hemin, and 1% vitamin K) was used as negative control (*n* = 5).

A non-targeted LC-MS based metabolomics approach was used to investigate the metabolite composition of CMGS and CMs (CMGS after *Clostridium* enrichment). A total of 1663 features were detected by all four streams of LC-MS analyses, i.e., positive and negative ionization modes with C18 and HILIC chromatography, in five soil CMs and CMGS. PCA was used to obtain 2D-PCA score plots visualizing the spatial distribution of soil CMs and CMGS medium. The first principal component (PC1) and the second principal component (PC2) in C18 negative data set accounted for 43.1 and 12.2% of the overall variability. PC1 and PC2 components explained 38.5 and 13.4%, 34 and 13.7%, and 37.1 and 10.8% of the total variability in C18 positive, HILIC negative and HILIC positive data sets, respectively. C18 and HILIC PCA score plots of both ionization modes showed a clear separation between CMGS and soil CMs (Figure 6). Farms 1, 3, and 5 clustered together in both C18 and HILIC PCA score plots showing less variability among samples in terms of metabolite composition. However, metabolite composition of F4SCM was found to be distinct from all other samples in both C18 and HILIC negative and positive ionization mode plots. F2SCM also showed a distinctive metabolite composition in C18 negative, C18 positive and HILIC positive ionization mode plots (Figure 6). To further verify the variation among samples based on the metabolite features, hierarchical clustering was carried out, which demonstrated a clear distance between F4SCM and rest of the samples (Supplementary Figures 1–4).

### Evaluation of Differentiating Metabolites of Farm 4 Soil Conditioned Medium (F4SCM) From Enrichment Medium (CMGS)

**FIGURE 7:**
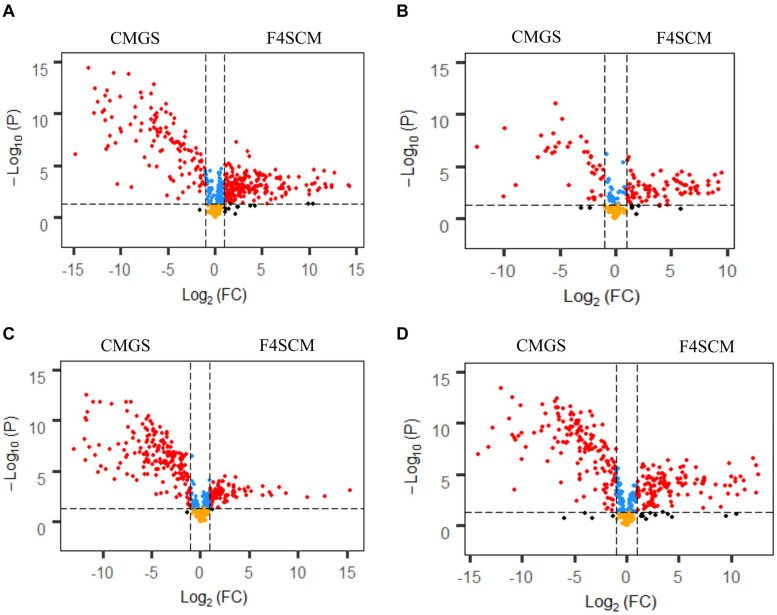
Volcano plots show the relative abundance of metabolite features by combining the statistical *t*-test [–log10(*p*-value)] and the magnitude of the change (log2 [FC]) in C18 negative **(A)**, C18 positive **(B)**, HILIC negative **(C)**, and HILIC positive **(D)** ionization mode datasets. Red dots represent the metabolites with *p*-value < 0.05 and FC > 2. Blue points represent the metabolites with *p* < 0.05 and FC < 2. Yellow points represent the metabolites with *p*-value > 0.05 and FC < 2. Black dots represent the metabolites with *p* > 0.05 and FC > 2. CMGS (Cooked meat glucose starch media supplemented with 0.0005% yeast extract, 0.1% hemin, and 1% vitamin K), F4SCM (Farm 4 soil conditioned medium).

F4SCM demonstrated the highest relative antimicrobial activity and significantly different metabolite profiles from all other soil CMs. Therefore, F4SCM metabolite profiles were further evaluated to identify metabolite features that differentiate them from enrichment medium (CMGS). Metabolite features detected in F4SCM and CMGS medium (497 from C18 negative, 209 from C18 positive, 490 from HILIC negative, and 467 from HILIC positive) were used to compute volcano plots showing significantly discriminating metabolites in both groups (Figure 7). In the volcano plot, metabolite features with a fold change (FC) > 2 and *P* < 0.05 were considered significantly different. A total of 539 metabolite features (210 from C18 negative, 84 from C18 positive, 100 from HILIC negative, and 135 from HILIC positive) were found to be significantly higher in the F4SCM group compared to CMGS. Significantly higher metabolites in F4SCM group, which are likely to be associated with its antimicrobial activity are presented on the right square shown by red color dots in volcano plots (Figure 7).

**FIGURE 8:**
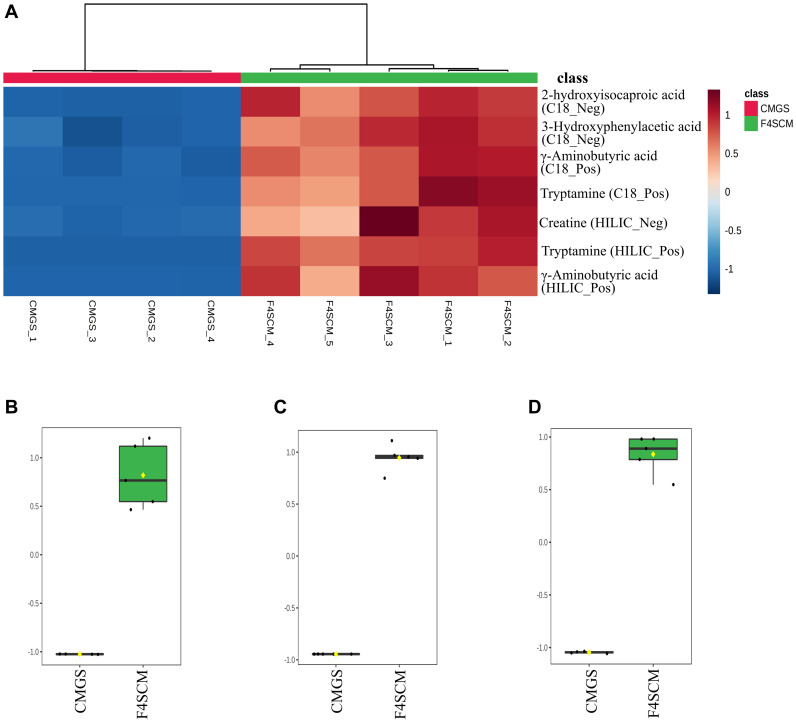
Intensity of putatively identified metabolites. **(A)** Heatmap of seven metabolites identified as significantly high in F4SCM. Rows represent putatively identified metabolites, whereas columns represent biological replicates. Metabolite intensity levels are shown using a pseudocolour scale (–1.0 to 1.0) with red representing high intensity levels and blue representing low intensity levels. **(B,C)** Plots represent the relative abundance of putatively identified tryptamine metabolite (normalized values ± SD) in C18 positive and HILIC positive datasets, respectively (*n* = 5). **(D)** Plot represents the relative abundance of putatively identified 2-hydroxyisocaproic acid (normalized values ± SD) in the C18 negative dataset (*n* = 5). CMGS (Cooked meat glucose starch media supplemented with 0.0005% yeast extract, 0.1% hemin, and 1% vitamin K), F4SCM- Farm 4 soil conditioned medium.

Significantly discriminating metabolite features in F4SCM were ranked using the *p*-values (lowest to highest) resulting in a prioritized list of tentative candidates based on the signal abundance. Supplementary Table 1 shows a list of top 50 significantly different metabolite features in F4SCM compared with CMGS from all four streams. Seven metabolites were identified with level two metabolite identification confidence as shown in Table 2. Supplementary Figures 5–11 provides information on extracted ion chromatograms for parent masses and co-eluting diagnostic fragments of matched compounds in the public MS database. Among seven identified metabolites, tryptamine and 2-hydroxyisocaproic acid were detected at very high levels in F4SCM group as shown in Figure 8.

**TABLE 2:** Putatively identified metabolite features in Farm 4 soil conditioned medium (F4SCM).

**Compound name**	**m/z**	**RT (sec)**	**Molecular formula**	**Stream**	**Adduct**	***p*-value**	**FDR**	**FC**	**Level of identification**
2-hydroxyisocaproic acid	131.0708	284.2	C_6_H_1__2_O_3_	C18_Neg	[M−H] −	1.65 × 10^–7^	9.83 × 10^–7^	19.3	2
3-Hydroxyphenylacetic acid	151.0395	295.3	C_8_H_8_O_3_	C18_Neg	[M−H] −	9.61 × 10^–7^	4.08 × 10^–6^	3.8	2
γ-Aminobutyric acid	104.0706	42.41	C_4_H_9_NO_2_	C18_Pos	[M+H]+	3.64 × 10^–7^	2.54 × 10^–6^	11.8	2
Tryptamine	161.1071	262.45	C_1__0_H_1__2_N_2_	C18_Pos	[M+H]+	1.14 × 10^–5^	4.42 × 10^–5^	427.0	2
Creatine	130.0612	723.24	C_4_H_9_N_3_O_2_	HILIC_Neg	[M−H] −	7.95 × 10^–5^	1.66 × 10^–4^	<2	2
Tryptamine	161.107	622.03	C_1__0_H_1__2_N_2_	HILIC_Pos	[M+H]+	8.20 × 10^–10^	6.84 × 10^–9^	7972.2	2
γ-Aminobutyric acid	104.0706	692.68	C_4_H_9_NO_2_	HILIC_Pos	[M+H]+	3.64 × 10^–7^	1.12 × 10^–6^	10.1	2

*m/z, mass to charge ratio; RT, retention time; p-value- t-test between F4SCM and CMGS groups; FDR, false discovery rate; FC, fold change value (F4SCM/CMGS).*

## Discussion

Microbial diversity provides opportunity for novel antimicrobial discovery. Microorganisms isolated from soil environments such as continental soils, desert soils, freshwater sediments, and marine sediments have been explored in the search for antimicrobials (Mullis et al., 2019). However, one of the most diverse groups of bacteria ubiquitously present in soil and other farm environments as well as in intestines of both animals and humans, *Clostridium* spp. has yet to be explored well for antimicrobial production (Walker, 1990; Pahalagedara et al., 2020).

In this study, we show that soil *Clostridium* enriched media present a potential source of antimicrobial compounds. The antimicrobial profiles of five soil *Clostridium* enriched media were evaluated against three well characterized and laboratory adapted bacterial reference strains, *B. cereus* NZRM 5, *B. mycoides* ATCC 6462, and *P. aeruginosa* ATCC 25668, representing pathogenic and food spoilage bacteria. The initial screening study revealed that all five soil CMs were active against the three tested microorganisms. Although nisin is an extensively used lantibiotic used in food preservation, it shows weak inhibition against Gram-negative bacteria (Lee et al., 2015; Li et al., 2018). Our study demonstrated that soil *Clostridium* enriched conditioned medium was effective in reducing/controlling the growth of *P. aeruginosa*, which has become a real concern in hospital acquired infections due to the appearance of multi-drug resistant strains. More in depth studies, need to be carried out to understand the activity of F4SCM against multi-drug resistant strains and the mechanism of action. Overall, F4SCM was found to be an attractive candidate for further investigations in the search for potent antimicrobials.

The putative active compound/s present in F4SCM against *P. aeruginosa* were thermostable and insensitive to protease enzyme indicating that they may not be proteinaceous in nature. On the other hand, putative antimicrobial/s in F4SCM inhibiting *B. mycoides* were heat labile at higher temperatures (70°C for 10 min or higher) but resistant to protease enzyme treatment, showing their differences in physiochemical nature from those against *P. aeruginosa*. Antimicrobial activity of F4SCM against *B. cereus* was influenced by both heat and protease enzyme segregating those presumptively active compound/s from others. F4SCM’s activity against all three tested microorganisms was partially affected by pH when deviated from its original pH (7.4 ± 0.2). These data suggest that F4SCM may contain different active compounds having diverse physiochemical characteristics. Moreover, these putative compounds may be providing synergistic antimicrobial activity.

16S rRNA gene sequence analyses confirmed that sequences of all farm 4 bacterial isolates were closely related to the genus *Clostridium*. Spiking sterile Farm 4 soil with different *Clostridium* spp. isolated from the same soil indicated the involvement of identified *Clostridium* isolates in F4SCM’s antimicrobial activity. To the best of our knowledge, this is the first report demonstrating the antimicrobial activity of soil *Clostridium* enriched medium. Further studies need to be carried out to investigate the antimicrobial potential of individual bacteria isolated from Farm 4 soil sample, using genomic and culture-based approaches to determine each micro-organism’s contribution to the antimicrobial activity.

Metabolomics is a high-throughput analytical technique, enabling the identification of low molecular weight metabolites in complex mixtures such as the conditioned media (French et al., 2018; Fudyma et al., 2019; Tang et al., 2020). In this study, we focused on secondary metabolites produced during *Clostridium* enrichment and their prospective contribution to antimicrobial activity. Non-targeted LC-MS analyses using C18 and ZIC-pHILIC columns were used to detect metabolites over a wide polarity range. PCA revealed the distinctive changes in metabolic composition of all five soil CMs showing the effect of *Clostridium* enrichment. F4SCM produced different polar and intermediate-polar metabolite profiles from those of other soil CMs and CMGS. This may explain the stronger antimicrobial activity of F4SCM than other soil CMs.

The comparison between F4SCM and CMGS metabolite profiles identified 539 significantly abundant metabolite features including some unique features in the F4SCM group suggesting its specialized chemical diversity. Both C18 and HILIC positive ionization modes revealed the presence of tryptamine in high abundance in the F4SCM group. Gut microorganisms such as *Clostridium* spp. and *Ruminococcus* spp. have been reported to produce tryptamine from tryptophan (Williams et al., 2014; Saraf et al., 2017). Tryptamine serve as precursors for complex clinically important alkaloid natural products including the neurotransmitters serotonin and psilocybin making them a medically significant group of molecules (Fricke et al., 2017). This group of compounds are reported to have growth inhibitory activities against some bacteria, fungi, and yeast. Chandrika and Ian (2020) demonstrated antibacterial and antifungal properties of tryptamine against *Saccharomyces cerevisiae*, *Candida krusei*, and *Candida tropicalis*. Tryptamine and its derivative, 6-bromo-8,10-dihydro-isoplysin A, extracted from a marine sponge *Fascaplysinopsis reticulata*, exhibited promising antibacterial activity against *Vibrio carchariae* and *Vibrio natrigens*, respectively (Campos et al., 2019). Conceivably, tryptamine may contribute to the antimicrobial property of F4SCM.

Another highly abundant putative metabolic compound in F4SCM group was identified as 2-hydroxyisocaproic acid (HICA), a product of leucine metabolism and a typical constituent of human plasma (Hoffer et al., 1993; Sakko et al., 2012). Fermentative bacteria such as lactic acid bacteria and some *Clostridium* spp. were reported to produce HICA using animal proteins. Brooks et al. (1984) reported that *Clostridium difficile* produced increased amounts of HICA in trypticase yeast salt broth enriched with isoleucine. HICA has been reported to show promising antimicrobial activity against pathogenic bacteria and fungi (Sakko et al., 2012, 2014). Sakko et al. (2012) investigated the antibacterial efficacy of HICA against a range of Gram-positive and Gram-negative bacteria including multi-drug resistant bacteria and found it highly effective against all tested bacteria at a concentration of 36 mg/mL. According to their study, HICA was highly active against *P. aeruginosa* with minimum bactericidal concentration (MBC) of 4.5 mg/mL. In the present study, we observed a strong growth inhibition of *P. aeruginosa* by F4SCM, that may be due to the effect of HICA produced by soil *Clostridium* isolates in animal proteins rich CMGS medium. However, further studies are required to confirm its contribution to the antimicrobial activity of F4SCM.

Other annotated metabolites in the F4SCM group were also related to the protein/amino acid metabolic pathways indicating the activation of protein metabolism by *Clostridium* enrichment. 3-Hydroxyphenylacetic acid is a monocarboxylic acid and it is a substrate for an enzyme in the tyrosine metabolism pathway (National Center for Biotechnology Information., 2020). Gamma-aminobutyric acid (GABA) is synthesized by decarboxylation of glutamate and produced by various groups of bacteria, particularly lactic acid bacteria (Kim et al., 2018).

Conceivably, individual isolates may have different levels of contribution to the total activity of F4SCM under the given growth conditions. Hence, it will be interesting to study the antimicrobial potential of each *Clostridium* isolate grown under various conditions such as mono and co-cultures. Moreover, genome analysis of *Clostridium* isolates may provide information on their genetic capability to synthesize potent antimicrobial compounds. Another extension to this work is investigating antimicrobial potential of clostridial antimicrobials against multi-drug resistant and true pathogenic bacteria. Our future work will include investigating the antimicrobial potential of F4SCM against different strains of bacterial species tested in this study, as different strains may behave differently to the antimicrobials present in F4SCM. This study reveals soil *Clostridium* spp. as a potential source of antimicrobial compounds and provides future prospects for antimicrobial discovery within their metabolic diversity against detrimental microorganisms associated with human health and food spoilage.

Limitation of this study includes incomplete characterization of metabolite profiles in the soil conditioned media due to limited mass spectral information in the currently available databases. Bioactivity-guided and targeted metabolomics studies are required to identify and characterize currently unknown metabolites present in soil conditioned media.

## Data Availability Statement

The original contributions presented in the study are included in the article/Supplementary Material, further inquiries can be directed to the corresponding author/s.

## Author Contributions

TG and AP designed the study. AP carried out most of the experiments and data analysis. AP and AS performed non-targeted metabolomics analyses. AP and TG wrote the manuscript with valuable feedback from SF, JP, AS, and GB. TG and GB attained funding for this project. All authors contributed to the article and approved the submitted version.

## Conflict of Interest

The authors declare that the research was conducted in the absence of any commercial or financial relationships that could be construed as a potential conflict of interest.

## Footnotes

1 http://rdp.cme.msu.edu/index.jsp

2 https://www.metaboanalyst.ca

## Abbreviations

CMs/CM: Conditioned media/Conditioned medium
F1SCM: Farm 1 soil conditioned medium
F2SCM: Farm 2 soil conditioned medium
F3SCM: Farm 3 soil conditioned medium
F4SCM: Farm 4 soil conditioned medium
F5SCM: Farm 5 soil conditioned medium
CMGS: Cooked meat glucose starch medium supplemented with yeast extract (0.0005%)hemin (0.1%) and vitamin K (1 %).
