# Supplementary material for: Antimicrobial Activity of Soil Clostridium Enriched Conditioned Media Against Bacillus mycoides, Bacillus cereus, and Pseudomonas aeruginosa
Source: Front Microbiol. 2020 Dec 4;11:608998. doi: 10.3389/fmicb.2020.608998 (PMC7746556; doi:10.3389/fmicb.2020.608998)
Supplement: Supplementary file 1 [file Data_Sheet_1.pdf]

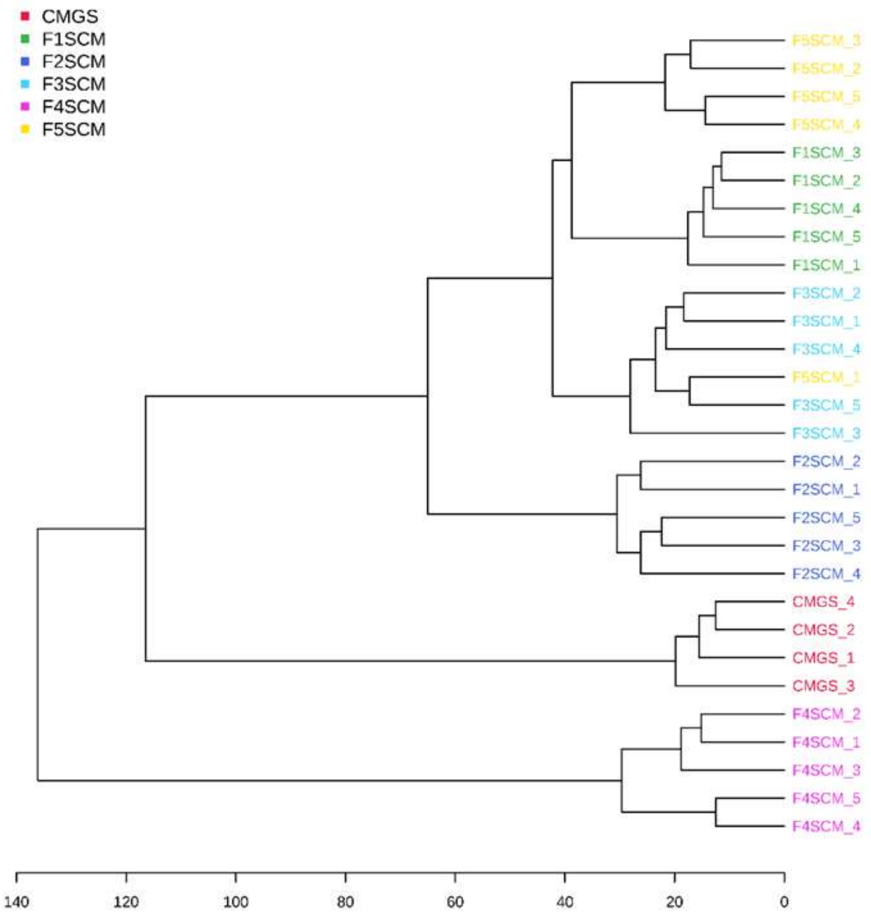

Supplementary Figure 1. Dendrogram showing the relationship between five soil CMs and CMGS medium based on their C18 negative ionization metabolite profiles. Dendrogram was constructed by ward clustering on the closest Euclidean distances between samples. Numbers represent sample ID.

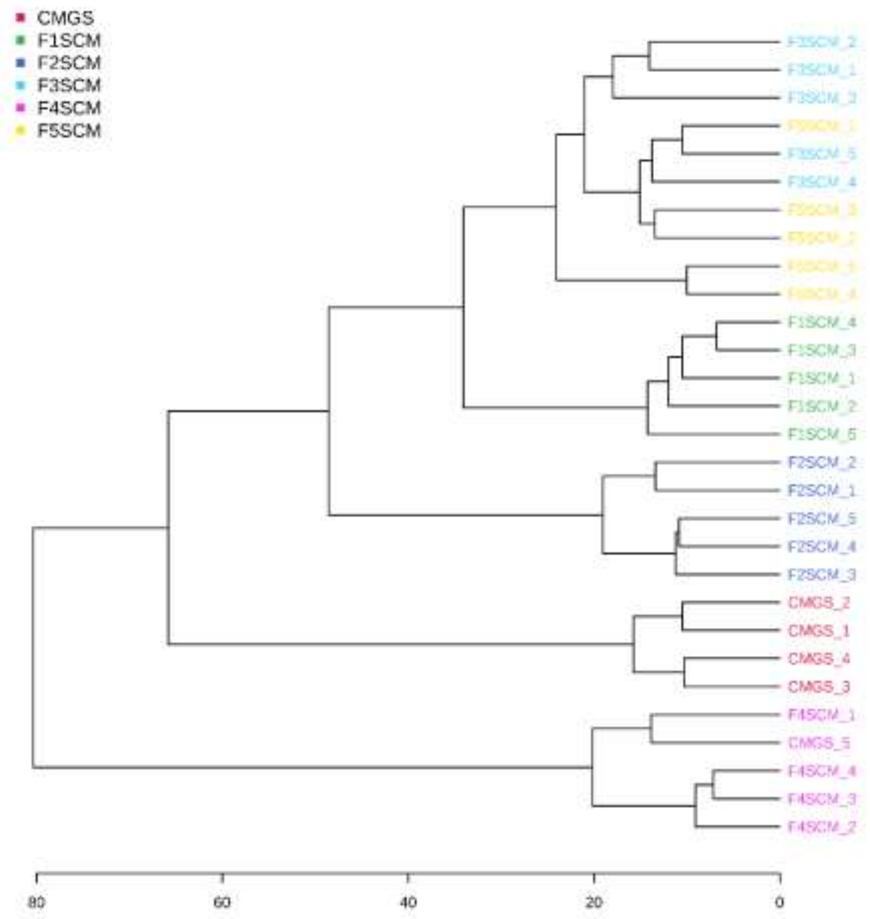

Supplementary Figure 2. Dendrogram showing the relationship between five soil CMs and CMGS medium based on their C18 positive ionization metabolite profiles. Dendrogram was constructed by ward clustering on the closest Euclidean distances between samples. Numbers represent sample ID.

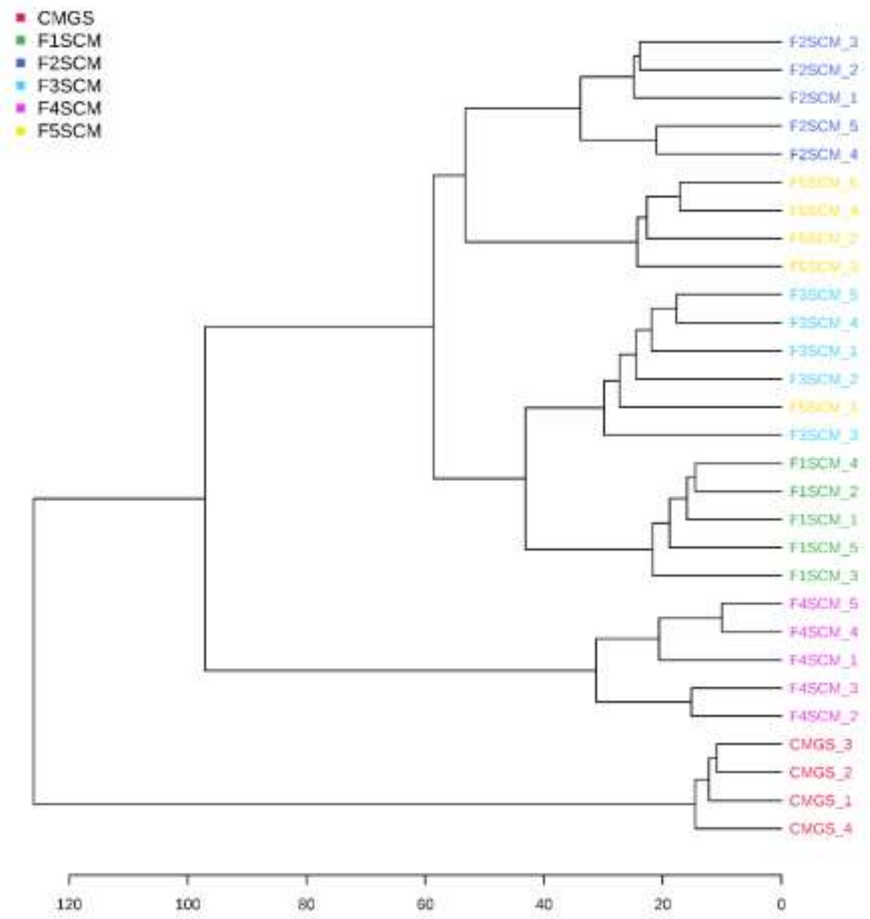

Supplementary Figure 3. Dendrogram showing the relationship between five soil CMs and CMGS medium based on their HILIC negative ionization metabolite profiles. Dendrogram was constructed by ward clustering on the closest Euclidean distances between samples. Numbers represent sample ID.

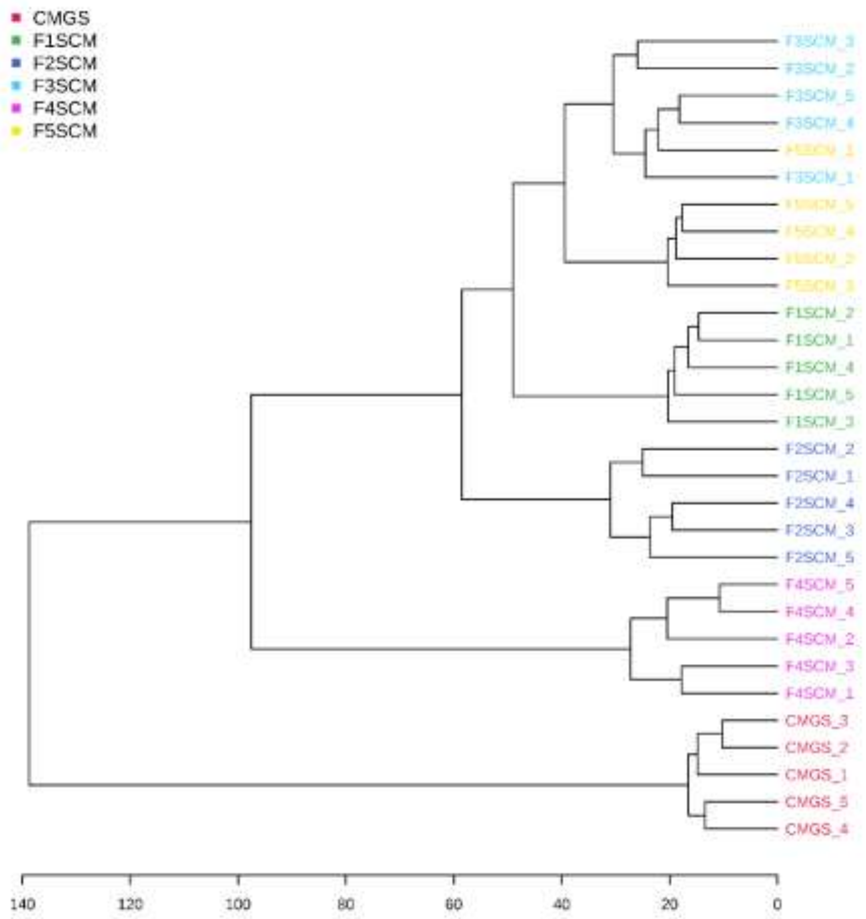

Supplementary Figure 4. Dendrogram showing the relationship between five soil CMs and CMGS medium based on their HILIC positive ionization metabolite profiles. Dendrogram was constructed by ward clustering on the closest Euclidean distances between samples. Numbers represent sample ID.

Compound name	m/z	RT (sec)	Molecular formula	Adduct	Fragments (*m/z*)	Level of identification
2-hydroxyisocaproic acid	131.0708	284.2	C6H12O3	[M-H]-	85.0662	2

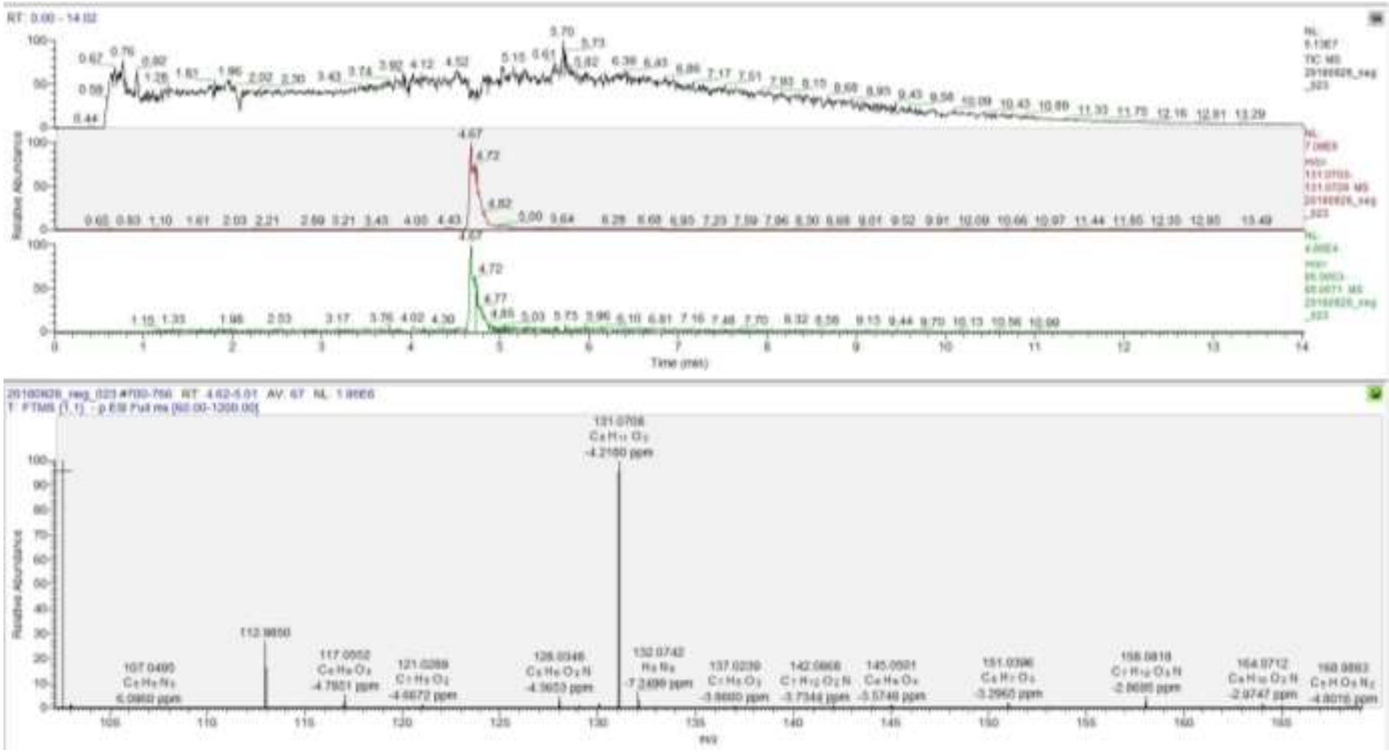

Supplementary Figure 5. Extracted ion chromatograms (C18 negative ionization) for parent masses and co-eluting diagnostic fragments of 2-hydroxyisocaproic acid in the public MS database.

Compound name	m/z	RT (sec)	Molecular formula	Adduct	Fragments (*m/z*)	Level of identification
3-Hydroxyphenylacetic acid	151.0395	295.3	C8H8O3	[M-H]-	107.0504	2

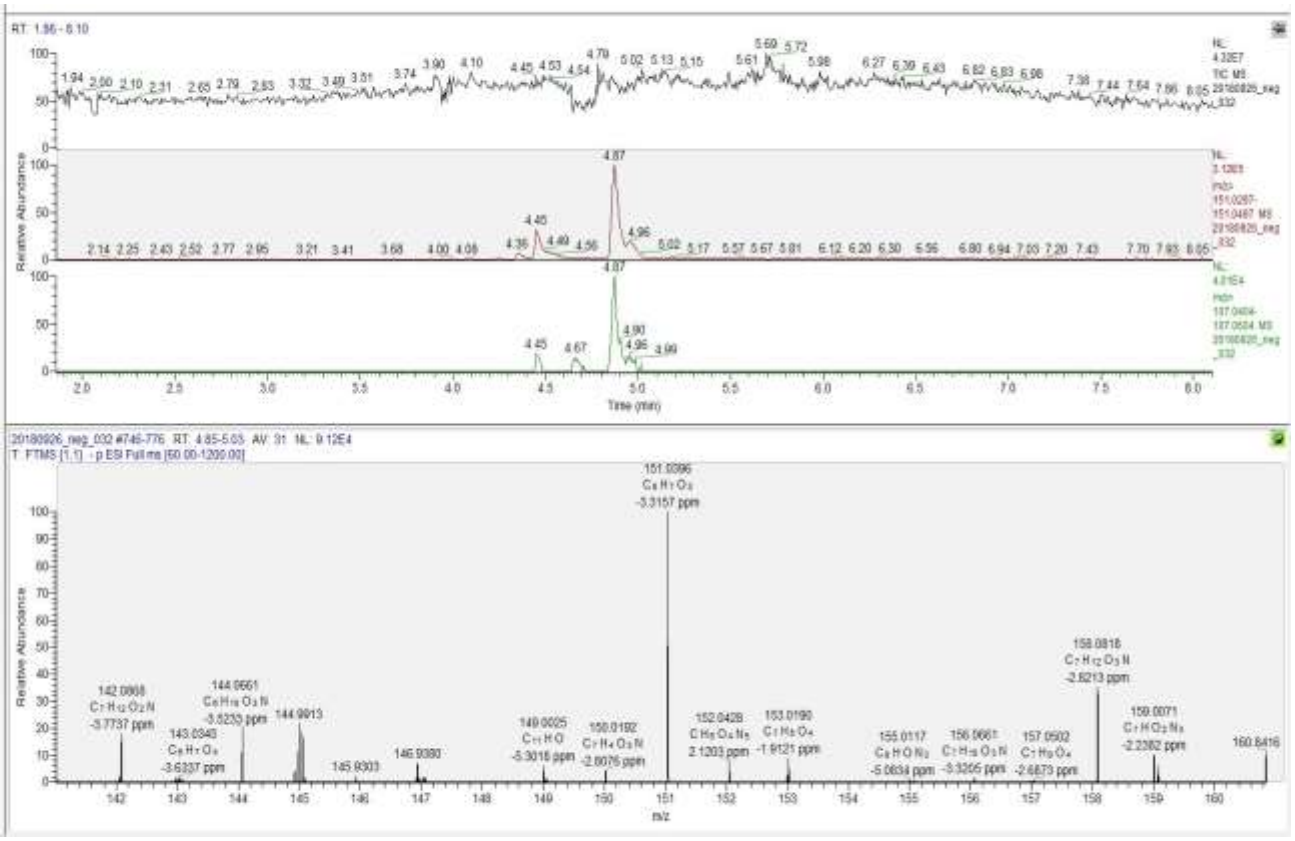

Supplementary Figure 6. Extracted ion chromatograms (C18 negative ionization) for parent masses and co-eluting diagnostic fragments of 3-hydroxyphenylacetic acid in the public MS database.

Compound name	m/z	RT (sec)	Molecular formula	Adduct	Fragments (*m/z*)	Level of identification
γ-Aminobutyric acid	104.0706	42.41	C4H9NO2	[M+H]+	87.0443, 86.0603	2

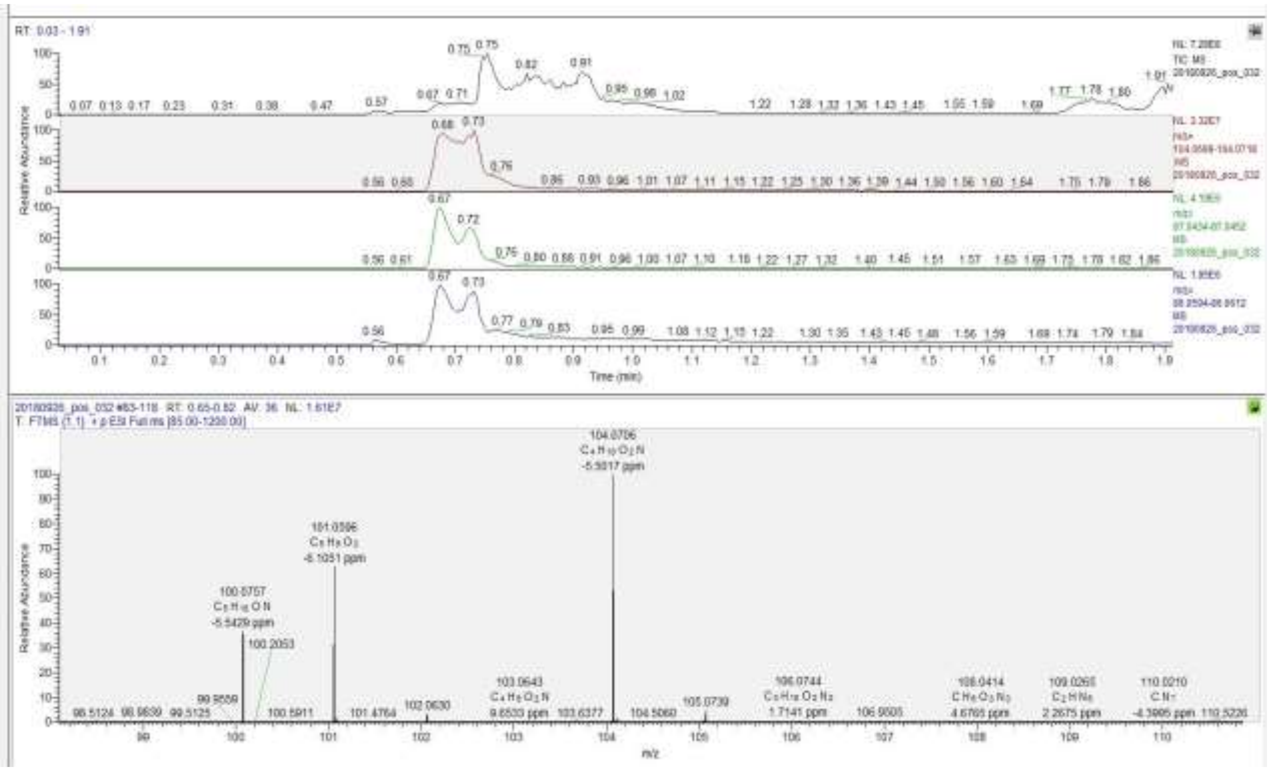

Supplementary Figure 7. Extracted ion chromatograms (C18 positive ionization) for parent masses and co-eluting diagnostic fragments of γ-aminobutyric acid in the public MS database.

Compound name	m/z	RT (sec)	Molecular formula	Adduct	Fragments (*m/z*)	Level of identification
Tryptamine	161.1071	262.45	C10H12N2	[M+H]+	144.0808	2

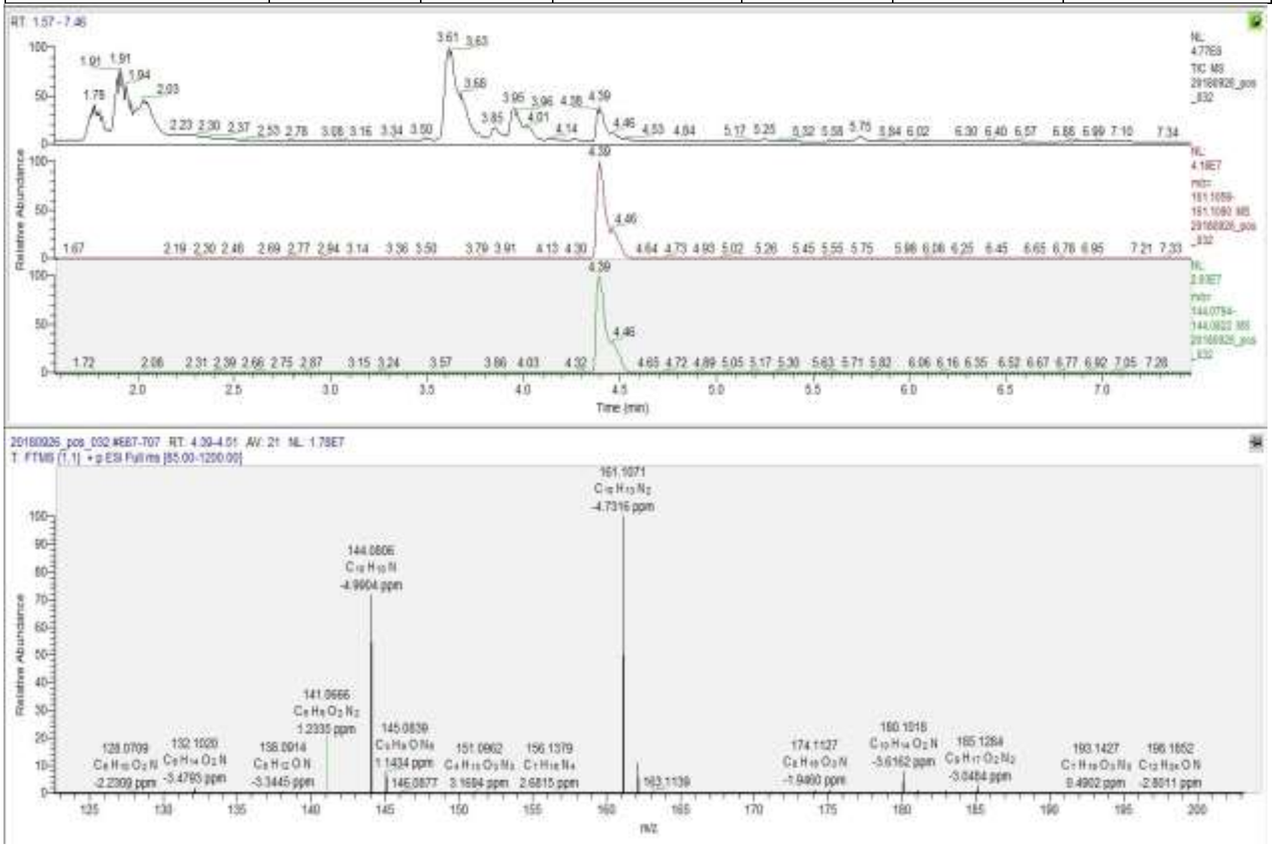

Supplementary Figure 8. Extracted ion chromatograms (C18 positive ionization) for parent masses and co-eluting diagnostic fragments of tryptamine in the public MS database.

Compound name	m/z	RT (sec)	Molecular formula	Adduct	Fragments (*m/z*)	Level of identification
Creatine	161.1071	723.24	C4H9N3O2	[M-H]-	88.0401	2

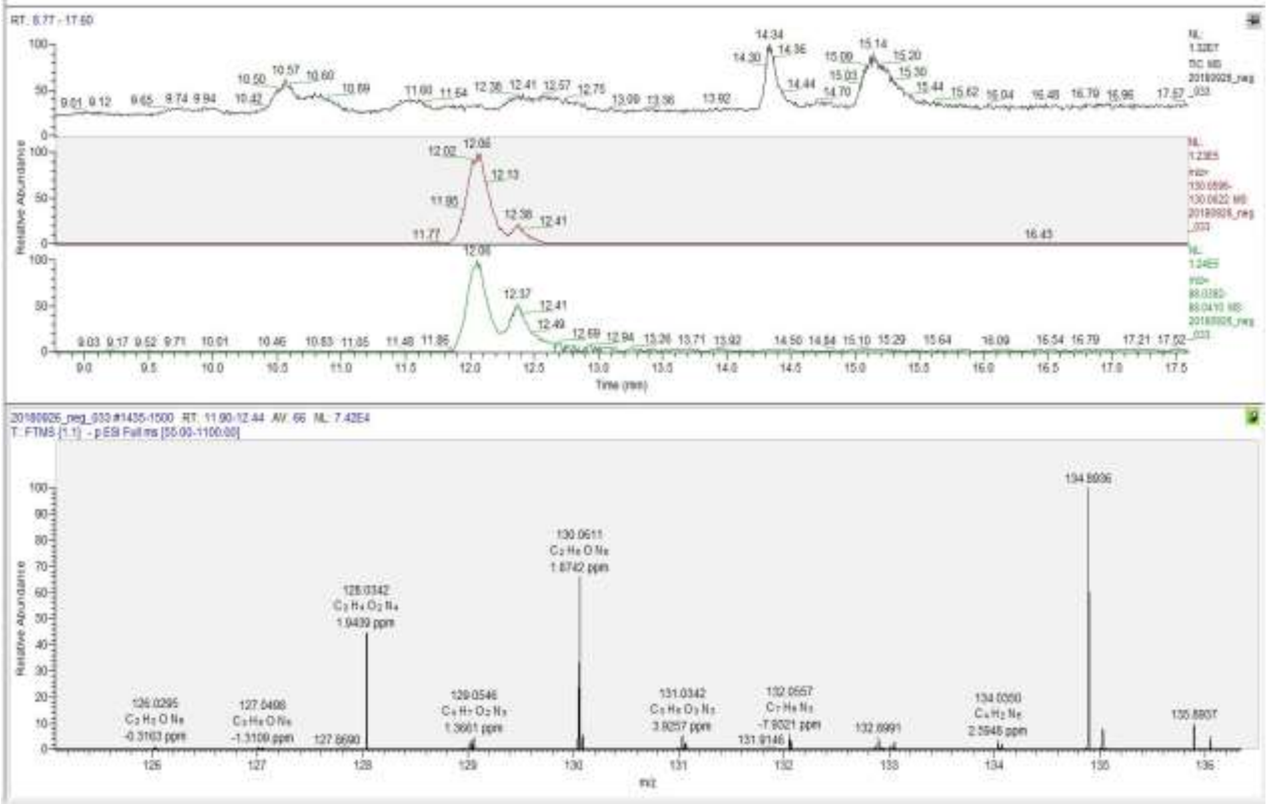

Supplementary Figure 9. Extracted ion chromatograms (HILIC negative ionization) for parent masses and co-eluting diagnostic fragments of creatine in the public MS database.

Compound name	m/z	RT (sec)	Molecular formula	Adduct	Fragments (*m/z*)	Level of identification
Tryptamine	161.107	622.03	C10H12N2	[M+H]+	427.0	2

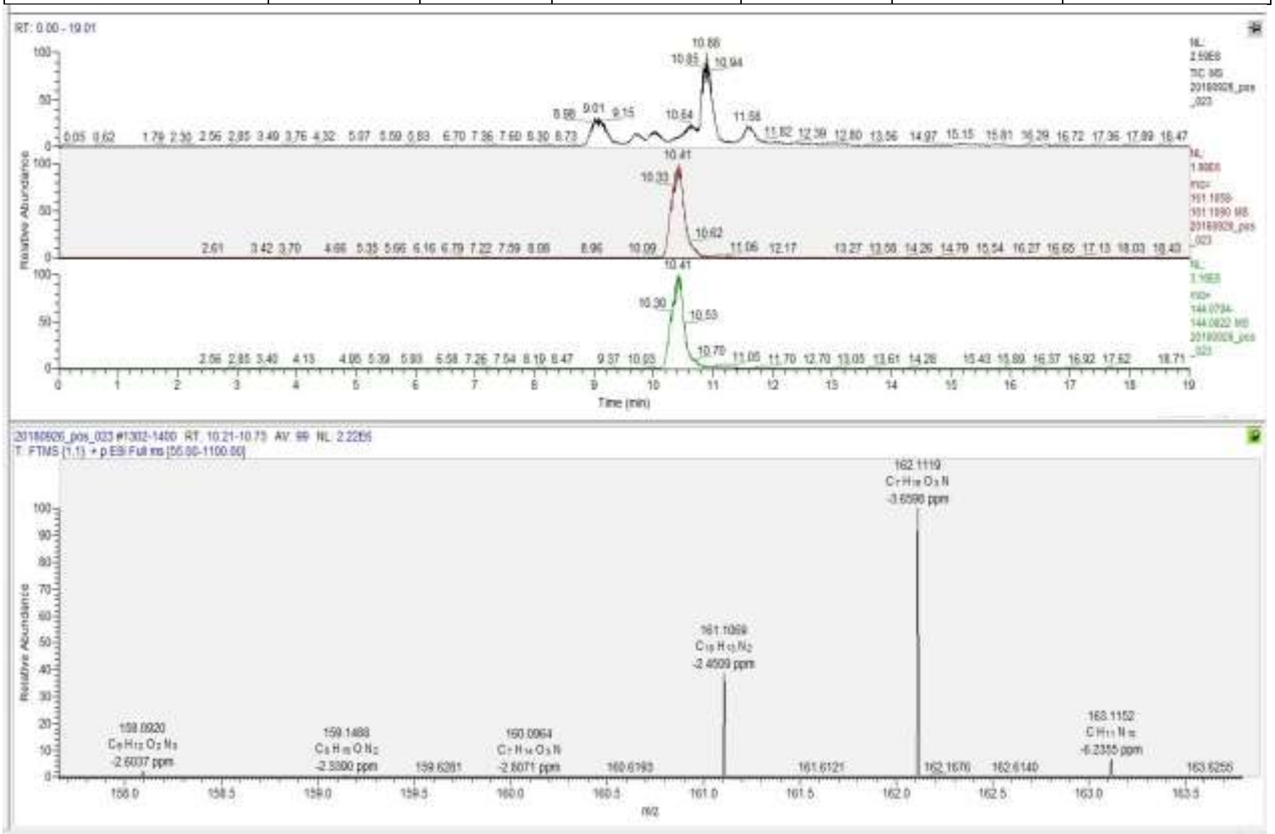

Supplementary Figure 10. Extracted ion chromatograms (HILIC positive ionization) for parent masses and co-eluting diagnostic fragments of tryptamine in the public MS database.

Compound name	m/z	RT (sec)	Molecular formula	Adduct	Fragments (*m/z*)	Level of identification
γ-Aminobutyric acid	104.0706	692.68	C4H9NO2	[M+H]+	87.0443, 86.0603, 69.0343	2

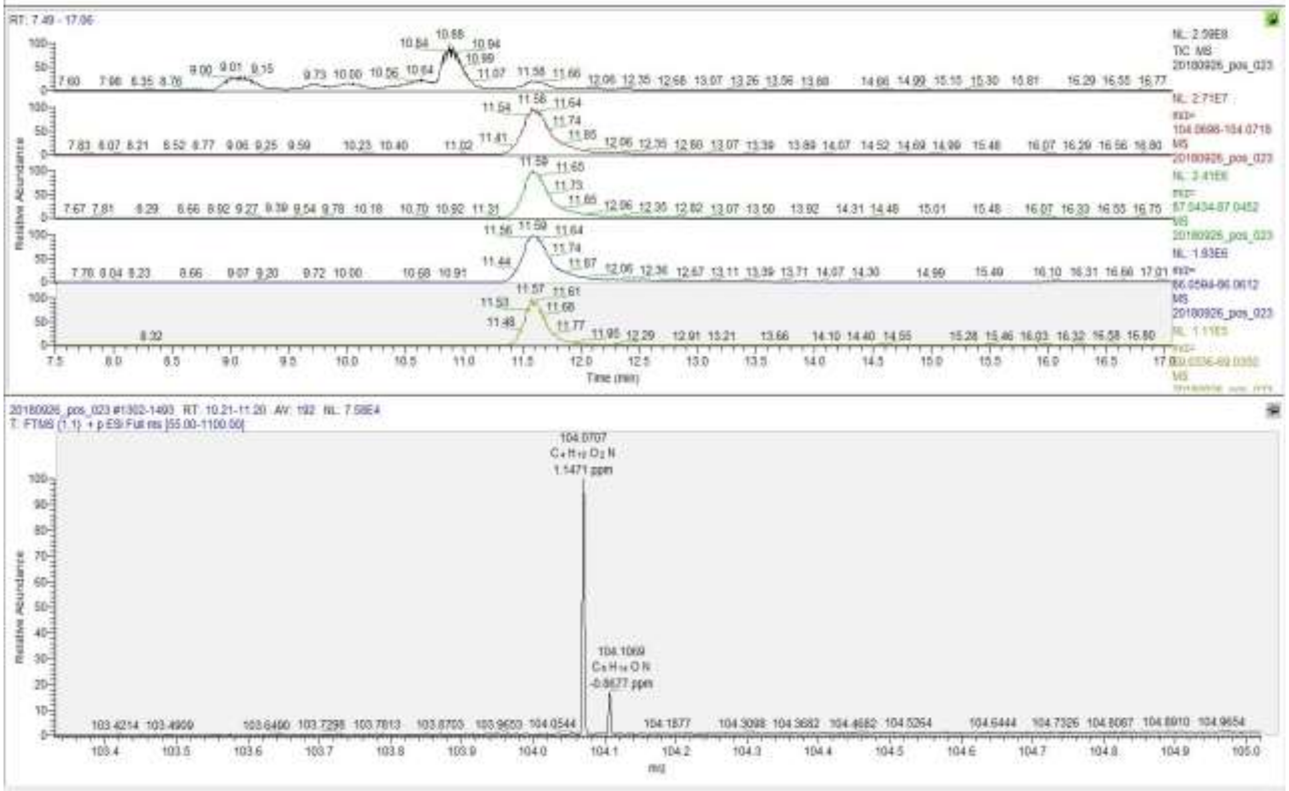

Supplementary Figure 11. Extracted ion chromatograms (HILIC positive ionization) for parent masses and co-eluting diagnostic fragments of γ-aminobutyric acid in the public MS database.

Supplementary Table 1. Significantly high metabolite features in F4SCM group (top 50 features ranked based on the *p*-value).

	C18 negative ionization				C18 positive ionization				HILIC negative ionization				HILIC positive ionization			
	m/z	RT	*p*-value	FC	*m/z*	RT	*p*-value	FC	*m/z*	RT	*p*-value	FC	*m/z*	RT	*p*-value	FC
**01**	172.0974	282.38	$2.39X10^{-10}$	5.4	180.9041	37.57	$4.52X10^{-9}$	2.3	374.1563	691.87	$1.75X10^{-7}$	6.0	161.107	622.03	$8.20X10^{-10}$	7972.2
**02**	362.9675	344.83	$3.34X10^{-10}$	11.9	142.9482	37.5	$1.33X10^{-8}$	2.2	336.1193	697.84	$2.00X10^{-7}$	13.0	72.0811	629.24	$1.41X10^{-9}$	60.7
**03**	131.0344	238.75	$2.15X10^{-9}$	18.9	126.0524	40.93	$1.06X10^{-7}$	15.3	261.0724	694.38	$8.29X10^{-7}$	4.5	274.1657	622.65	$1.77X10^{-9}$	13.8
**04**	61.9878	380.41	$5.35X10^{-8}$	2.2	164.9301	37.27	$1.29X10^{-7}$	2.8	245.125	869.8	$8.67X10^{-7}$	5.0	141.0653	632.7	$3.80X10^{-9}$	850.6
**05**	227.1399	267.28	$5.91X10^{-8}$	3.1	89.1075	36.04	$1.63X10^{-7}$	77.5	302.1352	690.07	$9.53X10^{-7}$	3.7	143.0686	632.55	$5.99X10^{-9}$	3.0
**06**	374.1566	247.69	$6.22X10^{-8}$	3.4	141.0656	55.86	$2.10X10^{-7}$	763.2	261.0877	681.74	$1.36X10^{-6}$	2.7	92.0708	861.87	$6.53X10^{-9}$	6.2
**07**	298.1517	209.11	$6.42X10^{-8}$	2.2	104.0706	42.41	$3.64X10^{-7}$	11.8	188.1033	781.06	$2.66X10^{-6}$	8.4	123.0553	632.58	$7.29X10^{-9}$	5452.1
**08**	390.1158	60.69	$9.61X10^{-8}$	3.8	177.0741	343.31	$3.93X10^{-7}$	343.31	242.1138	886.45	$3.08X10^{-6}$	6.5	167.0812	657.18	$8.25X10^{-9}$	3.3
**09**	250.0574	362.26	$1.10X10^{-7}$	103.2	176.0707	343.59	$4.13X10^{-7}$	111.2	258.0724	742.65	$3.28X10^{-6}$	11.3	81.0449	632.74	$1.02X10^{-8}$	9.3
**10**	256.0646	278.02	$1.15X10^{-7}$	1493.9	188.1758	39.64	$4.41X10^{-7}$	50.2	189.0509	695.32	$3.49X10^{-6}$	3.4	100.0758	651.25	$1.18X10^{-8}$	112.2
**11**	380.1825	290.49	$1.29X10^{-7}$	3.1	148.0343	41.03	$6.92X10^{-7}$	3.5	166.0172	591.4	$4.13X10^{-6}$	3.7	144.0805	622.42	$1.20X10^{-8}$	37.1
**12**	243.1348	240.69	$1.35X10^{-7}$	11.3	202.144	343.19	$1.68X10^{-6}$	42.7	61.9873	633.62	$4.55X10^{-6}$	3.5	131.1178	655.11	$1.27X10^{-8}$	2.8
**13**	107.0496	295.05	$1.36X10^{-7}$	4.9	137.0267	61.99	$2.09X10^{-6}$	3.0	204.0619	750.87	$4.58X10^{-6}$	12.4	59.0493	650.66	$2.01X10^{-8}$	20.0
**14**	131.0708	284.2	$1.65X10^{-7}$	19.3	208.1335	314.05	$2.85X10^{-6}$	314.8	232.0819	692.87	$4.60X10^{-6}$	40.1	181.1043	550.47	$4.02X10^{-8}$	4.0
**15**	102.0553	214.63	$1.66X10^{-7}$	3106.1	184.1334	343.01	$3.26X10^{-6}$	46.0	141.0537	634.1	$4.71X10^{-6}$	88.8	87.0443	692.59	$7.12X10^{-8}$	11.4
**16**	181.0362	207.4	$2.39X10^{-7}$	13.7	199.144	214.91	$3.31X10^{-6}$	48.4	139.0501	634.1	$5.47X10^{-6}$	313.8	275.123	384.19	$7.63X10^{-8}$	8.3
**17**	408.1411	259.96	$2.64X10^{-7}$	9.5	131.1177	45.81	$3.83X10^{-6}$	4.7	102.0186	324.23	$8.06X10^{-6}$	2.0	86.0603	692.66	$8.38X10^{-8}$	10.7
**18**	131.0344	225.55	$2.72X10^{-7}$	29.2	264.1595	416.39	$4.38X10^{-6}$	115.7	227.1143	875.22	$8.55X10^{-6}$	3.9	180.1014	550.23	$1.12X10^{-7}$	5289.9
**19**	101.0601	313.44	$2.93X10^{-7}$	6818.2	115.0504	93.81	$4.62X10^{-6}$	21.3	175.024	502.39	$9.42X10^{-6}$	4.2	68.0497	692.68	$1.26X10^{-7}$	57.8
**20**	213.0878	256.96	$3.93X10^{-7}$	2.5	144.0806	262.44	$4.65X10^{-6}$	798.8	167.9963	854.34	$1.16X10^{-5}$	3.6	245.1492	591.97	$1.48X10^{-7}$	13.1
**21**	85.0655	283.37	$4.14X10^{-7}$	9.3	177.1027	231.18	$5.81X10^{-6}$	59.2	152.0567	647.54	$1.20X10^{-5}$	17.9	82.0652	650.63	$1.64X10^{-7}$	1228.0
**22**	132.0742	282.84	$4.32X10^{-7}$	19.8	133.0972	35.41	$7.56X10^{-6}$	2.6	187.108	745.98	$1.24X10^{-5}$	3.6	118.086	649.96	$1.72X10^{-7}$	4.4
**23**	193.9946	268.55	$4.57X10^{-7}$	28.7	105.0739	42.5	$8.56X10^{-6}$	4.6	155.0817	650.65	$1.30X10^{-5}$	20.8	61.0844	597.12	$1.85X10^{-7}$	2892.2
**24**	346.198	273.22	$5.30X10^{-7}$	90.1	100.0756	214.9	$1.07X10^{-5}$	30.7	96.9686	747.63	$1.31X10^{-5}$	2.2	119.0895	650.13	$1.93X10^{-7}$	4.6
**25**	345.1499	242.3	$5.62X10^{-7}$	5.3	229.1551	203.66	$1.09X10^{-5}$	6.8	95.0604	634.1	$1.43X10^{-5}$	41043.0	148.0964	690.6	$2.40X10^{-7}$	4.8
**26**	279.0984	251.61	$5.78X10^{-7}$	2.4	101.079	214.83	$1.11X10^{-5}$	73.6	299.1145	651.04	$1.46X10^{-5}$	11.9	245.1491	558.09	$2.65X10^{-7}$	12.5
**27**	175.0243	116.1	$6.38X10^{-7}$	37.8	161.1071	262.45	$1.14X10^{-5}$	427.0	132.0293	692.36	$1.50X10^{-5}$	2.8	101.0597	650.39	$2.74X10^{-7}$	191.5
**28**	180.9993	312.17	$6.43X10^{-7}$	2.3	121.0648	122.53	$1.23X10^{-5}$	46.2	233.0232	739.66	$1.57X10^{-5}$	3.5	74.0715	654.79	$2.75X10^{-7}$	3.4
**29**	327.167	231.58	$6.65X10^{-7}$	4.4	170.0926	38.67	$1.63X10^{-5}$	8.6	257.1251	893.41	$1.72X10^{-5}$	5.0	105.074	692.58	$2.94X10^{-7}$	12.0
**30**	310.1406	272.94	$6.71X10^{-7}$	30.9	138.0913	122.62	$1.66X10^{-5}$	180.6	171.0403	693.86	$1.77X10^{-5}$	3.8	134.0957	550.08	$3.00X10^{-7}$	40.3
**31**	144.066	214.23	$8.88X10^{-7}$	23.1	376.1723	246.56	$1.68X10^{-5}$	3.4	431.1775	696.4	$2.00X10^{-5}$	29.4	56.0497	650.38	$3.23X10^{-7}$	32.7
**32**	231.0982	204.07	$8.94X10^{-7}$	10.8	102.0801	214.58	$2.07X10^{-5}$	7.8	121.0397	634.1	$2.05X10^{-5}$	26.3	93.0701	636.27	$3.48X10^{-7}$	1274.4
**33**	130.0867	344.67	$8.97X10^{-7}$	4.2	100.0488	214.72	$2.21X10^{-5}$	145.1	144.0657	650.53	$2.27X10^{-5}$	2.2	103.0504	741.82	$3.61X10^{-7}$	36.9
**34**	238.021	112.49	$9.10X10^{-7}$	32.0	247.1292	235.16	$2.36X10^{-5}$	2.4	129.0184	455.53	$2.39X10^{-5}$	2.9	104.0706	692.68	$3.64X10^{-7}$	10.1
**35**	151.0395	295.3	$9.61X10^{-7}$	3.8	132.102	343.31	$2.80X10^{-5}$	2.4	271.0568	785.47	$2.60X10^{-5}$	79.9	170.0808	581.53	$3.67X10^{-7}$	4.0
**36**	186.113	310.03	$1.03X10^{-6}$	145.6	174.1129	270.86	$2.89X10^{-5}$	120.4	206.9961	597.53	$2.62X10^{-5}$	12.0	188.1279	583.53	$3.70X10^{-7}$	5.4
**37**	179.0709	345.72	$1.16X10^{-6}$	289.0	154.0972	55.62	$3.43X10^{-5}$	455.6	214.035	695.33	$2.64X10^{-5}$	4.8	376.1706	691.48	$3.89X10^{-7}$	7.2
**38**	133.075	284.16	$1.22X10^{-6}$	3.4	230.1752	411.4	$3.55X10^{-5}$	52.7	146.0449	694.73	$2.68X10^{-5}$	3.0	289.0834	648.77	$3.91X10^{-7}$	478.0

	C18 negative ionization				C18 positive ionization				HILIC negative ionization				HILIC positive ionization			
	m/z	RT	*p*-value	FC	*m/z*	RT	*p*-value	FC	*m/z*	RT	*p*-value	FC	*m/z*	RT	*p*-value	FC
**39**	165.0551	326.7	$1.25X10^{-6}$	44.7	360.1925	288.38	$3.60X10^{-5}$	2.2	164.0014	648.7	$2.70X10^{-5}$	2.4	69.0338	692.48	$4.14X10^{-7}$	32.6
**40**	180.0741	345.69	$1.25X10^{-6}$	777.4	220.1006	304.23	$3.74X10^{-5}$	6.9	228.0983	652.23	$2.83X10^{-5}$	4.5	105.066	437.15	$4.33X10^{-7}$	6.5
**41**	382.1729	224.02	$1.28X10^{-6}$	11.7	194.1181	285.91	$3.76X10^{-5}$	23.4	207.0879	655.51	$3.11X10^{-5}$	10.5	139.0942	637.23	$4.76X10^{-7}$	112.1
**42**	336.12	254.66	$1.42X10^{-6}$	6.9	177.102	216.42	$3.81X10^{-5}$	11.7	227.1031	674.94	$3.16X10^{-5}$	3.9	121.0648	636.81	$4.94X10^{-7}$	188.5
**43**	262.1446	417.53	$1.58X10^{-6}$	106.0	188.1284	308.67	$4.48X10^{-5}$	109.4	130.0501	748.19	$3.42X10^{-5}$	4.9	76.076	664.05	$5.41X10^{-7}$	190.1
**44**	263.1481	417.51	$1.61X10^{-6}$	951.3	192.598	222.94	$4.94X10^{-5}$	9.1	147.0288	455.34	$3.69X10^{-5}$	3.0	55.0545	649.88	$6.01X10^{-7}$	6.7
**45**	149.0603	380.88	$1.73X10^{-6}$	107.6	190.0864	376.55	$5.10X10^{-5}$	142.0	171.0403	844.2	$3.71X10^{-5}$	3.3	138.0909	637.15	$7.04X10^{-7}$	181.6
**46**	344.1461	242.32	$1.77X10^{-6}$	101.4	360.2131	298.49	$5.15X10^{-5}$	8.4	279.1094	641.69	$3.72X10^{-5}$	2.4	405.0904	515.91	$7.13X10^{-7}$	3234.7
**47**	245.114	236.91	$1.88X10^{-6}$	2.4	141.0655	78.9	$6.44X10^{-5}$	12.7	403.0761	516.58	$3.84X10^{-5}$	338.7	229.1541	511.18	$8.04X10^{-7}$	13.3
**48**	288.1198	200.13	$2.05X10^{-6}$	6.3	155.0792	36.58	$7.34X10^{-5}$	3.0	127.039	650.04	$4.34X10^{-5}$	3.7	212.1025	529.08	$8.14X10^{-7}$	5.6
**49**	383.158	223.78	$2.06X10^{-6}$	7.7	318.1668	243.86	$1.13X10^{-4}$	2.0	215.0667	833.28	$4.69X10^{-5}$	2.6	145.133	639.18	$8.28X10^{-7}$	10.5
**50**	188.0347	253.01	$2.19X10^{-6}$	7.3	216.1596	382.5	$1.17X10^{-4}$	93.7	214.0827	689.38	$4.81X10^{-5}$	3.0	123.0998	601.36	$9.35X10^{-7}$	1553.7

m/z – mass to charge ratio

RT – retention time (seconds)

p-value - *t*-test between F4SCM and CMGS groups

FC – fold change value (F4SCM/CMGS)
